# Supplementary material for: Collapse and continuity: A multi-proxy reconstruction of settlement organization and population trajectories in the Northern Fertile Crescent during the 4.2kya Rapid Climate Change event
Source: PLoS One. 2021 Jan 11;16(1):e0244871. doi: 10.1371/journal.pone.0244871 (PMC7799814; doi:10.1371/journal.pone.0244871)
Supplement: Attachment — Submitted filename: Reviewer 1 Nov 18 2020.pdf [file pone.0244871.s003.pdf]

Reviewer 1

Red= reviewer 1 initial review

Black = authors' response to review

Green = Reviewers response to authors

1. Blurs high resolution 14C chronology in multiple regions into arbitrary 100 year bins, when the events and processes described are measurably visible at AMS-calibrated Oxcal-regulated and Bayes- manipulated decadal levels;

The technique used and described in the paper does not blur chronological dates and does not assign individual dates to 100 year bins. We use an established technique (see citations in the paper) to mitigate the impact of single site phases with multiple radiocarbon dates close together on our curves. This uses 50 year bins (not 100 year bins) and is only applied to sites where there are multiple C14 dates within 50 years of one another (as explained in lines 190-192). Where we have multiple dates within 50 years the uncalibrated radiocarbon age and error of all dates are averaged, and we then calibrate the averaged date rather than multiple dates. We want to stress that this technique is only applied to a small number of phases at a small number of sites where

Note that their figures, illustrating their data analysis, reduce abrupt period-wise changes in population to gradual, attenuated changes-- precisely opposite their claim, or as they state below, they "smooth fluctuations resulting from sampling biases….".

Although not explicitly stated, we understand the reviewer to be concerned that the binning technique we use will mask very short-term fluctuations in the curve which can be attributed to the effects of the 4.2k event. We hope that the fact that we are applying a 50 year rather than 100 year bin will mitigate this concern, along with the clarification that we are only applying this to a limited number of sites. More fundamentally, the effect on the dataset is not to blur or coarsen it, but to smooth fluctuations resulting from sampling biases (something which the reviewer raises as important in point 7 below). We would also note that at a regional scale the duration of the 4.2k event is considered to be centennial rather than decadal (as we state in lines 79-81, with citations), meaning the resolution of our C14 dataset should be sufficiently precise to identify fluctuations resulting from it. In fact, as we state in lines 348-350 and show in Figures 2 and 7, we can see a rapid decline at 4.2k over only 100 years. We argue in lines 351-355 that it is the increased precision of C14 dates relative to other proxies which allows us to 'see' the event in this dataset.

The authors' "rapid decline", the authors' claim, is in fact represented by a gradual curve, that masks the abrupt 95% abandonment of the Khabur Plains for more than 150 years as documented by high resolution radiocarbon dating of regional ceramic periodizations.

Pertinent to the authors' claims of "rapid" but "smoothed" decline is the archaeological observation of 4 major public building projects at sites across this region that were Akkadian initiated but abandoned unfinished by the Akkadians precisely at 4.2 event onset.

To judge from their century-scale settlement maps, the authors appear to be using regional and site settlement occupation data widely discredited (i.e., Oates' and McMahon "all of Brak was always inhabited"), cf. Emberling 2012, Arrivabeni 2012.

2. Misses Spain, France, Italy, Greece, Palestine, Egypt, Indus and East Asia societal collapses synchronous at 4.2 ka BP with northern Levant and northern Mesopotamia ("northern Fertile Crescent");

Our paper does not pretend to include all of the regions where the 4.2kya event has been considered to have an impact, and we state the location of the study region in the title, abstract and introduction. We also acknowledge that the event is considered to have impacted societies beyond out study region (lines 55-56, 85-88) and provide several citations to support this. As we state in lines 85-92, the goal of our study is to investigate the event at a regional scale using empirical data as opposed to synthetic comparison, and the area we have chosen is based on a shared physical geography. A future project might compare all of the regions described by the reviewer using archaeological data but this would require years if not decades of data collection. While this work is undertaken, we feel regional analyses can still advance the debate on this topic, as Reviewer 2 suggests.

The synchronous collapses in adjacent regions is hardly irrelevant--it confounds the attempt to explain the collapse in this region as due to one, albeit fictional, carrying capacity limit, as the adjacent regions, extending to Spain, had very different agro-production and population regimes, yet also were abandoned.

3. Misses westerlies, ISM, and EASM synchronous, high resolution, abrupt 4.2 ka BP event;

4. . Misses high resolution northern Levant and north Mesopotamian region proxy data for abrupt, century-scale, 4.2 ka BP event;

We are unsure what the reviewer is referring to here, but assume they mean climate proxy data. If this is the case then as above we stress that our paper is not addressing the climate proxy data, although we do cite several overview papers on climate proxy data derived from natural archives (speleotherms, pollen, formanifera etc) in the region. We have added text clarifying this in lines 82-84 and an additional date range in line 80. If the reviewer is referring to specific proxy data for another variable then we would be happy to address this concern if provided with more detail.

As stated in lines 66-68, the goal of this paper is to examine population and settlement organisation during the period of the 4.2kya event. We take it as fact that the event occurred in some form, and briefly explain the state of the debate on its magnitude and duration with appropriate citations (lines 71-82). As such we do not go into detail on the climate forcing mechanisms, such as the westerlies, Indian Summer Monsoon (ISM) and East Asian Summer Monsoon (EASM) shifts mentioned by the reviewer, which may account for the climate event itself. We are aware of these mechanisms, and cite relevant literature which deals with them, including our own work (Cookson et al. 2019), but we do not feel that further discussion is relevant to the goals of the paper. We have added text clarifying that we are not dealing with the climate proxies here in lines 82-84 and an additional date range in line 80.

The synchronous ISM and EASM region failures generated synchronous regional social collapses, which again are relevant to the claim that "carrying capacity", as here defined, was "exceeded." Was it exceeded synchronously everywhere? Or, nowhere?

The magnitude and duration of the 4.2 event seem immediately relevant to the authors' claim that it was not relevant to the 4.2 social collapses, especially if their claim that collapse was inevitable without it. Was it? Are their descriptions of it credible, recent, high resolution? It appears none of the above.

5. Takes inexpert, pop archaeology, opinions (e.g., Middleton), lacking any data, for facts of Akkadian collapse;

We cite multiple sources on the collapse of the Akkadian Empire, all of which come from peer reviewed journal articles, books and edited volumes. None of these would be considered popular archaeology. The two citations of Middleton are from a Cambridge University Press single author volume and a chapter in an edited volume. The CUP volume is not a popular science volume and was (favourably) reviewed in several academic publications including the European Journal of Archaeology (Smart 2018) and the American Journal of Archaeology (Tainter 2018) as an academic work. The chapter cited comes from a volume edited by Eustathios Chiotis which is also not a popular science book. All contributions to this volume were peer reviewed and the authors are all established academics. As such we feel this comment is unwarranted. We also note that the reviewer does not identify any substantive criticisms of our treatment of the Akkadian Empire in the paper. Without knowing what facts are incorrect we cannot amend the manuscript.

Cambridge Univ Press is not Cambridge University and like other major research university affiliated presses has moved to popular science books. Middleton is one such book's author.

"established academics"? Middleton has never had an academic appointment in his life. He teaches English-as-a second-language in Japan according to his own biography; he has never studied nor practiced Near Eastern archaeology, much less third millennium BC Mesopotamian archaeology and history. Citing him, inter alia, as an authority seems disingenuous, when he more credibly is described as a pop archaeologist.

The authors claim **"Ll 419-420: Unlike in earlier periods, such as the 420 Uruk, or later territorial empires** (sic!), **archaeologists cannot currently recognize clear ceramic 421 indicators directly and unambiguously associated with Akkadian presence (81).81. McMahon A. Post-Akkadian ceramic assemblages of the central Upper Khabur: What can 645 pottery tell us about political and climate change? In: Weiss H, editor. Seven Generations 646 since the Fall of Akkad. Wiesbaden: Harrassowitz. 2012: 25-44. This means we 422 cannot detect when or under what conditions Akkadian control in Upper Mesopotamia came to an ll 423 end."**

But multiple published authorities define, discriminate, illustrate and quantify Akkadian and, even, post-Akkadian ceramic assemblages, e.g., Arrivabeni, Ristvet, Quenet, Emberling, Ur, Gibson, Schmidt, Orsi, Matthews, Pfalzner, to mention the obvious published analyses.

"This means we cannot detect…."

But this is refuted by multiple lines of archaeological and epigraphic evidence abundantly published in the peer-reviewed archeological literature:

"…when Akkadian control in Upper Mesopotamia came to an end":

Weiss et al 2012, provides >100 14C dates, defining short ceramic periods, for ca. 1600 sq kms survey, with regional abandonment identified 2200-1950 BC, synchronous with Akkadian abandonments at Leilan Acropolis and Lower Town, Brak Acropolis and Lower Town, Mozan Acropolis and Lower Town, Moh. Diyab, Hamoukar, Taya, the first three also 14C dated and epigraphically identified, by multiple archaeologists..

"…under what conditions Akkadian control in Upper Mesopotamia came to an end": The expansions of Akkadian major public building constructions were abandoned abruptly, unfinished and precisely dated with high resolution radiocarbon dates, and epigraphically dated, across Upper Mesopotamia at Leilan, Mozan, Brak, Moh. Diyab, and Tell Taya, and similarly discussed in Weiss et al 2012.

6. Guesses at testing of regional societies' carrying capacity limit at end 3rd mill., when post 4.2 ka BP event settlement and population were both successful and multiple times greater across this region;

The carrying capacity limit is not a guess, it is a function of the logistic model for population growth we use as a null model. The model assumes that the long term growth rate of pre-industrial populations fits a logistic curve. The logistic population curve assumes population gradually increased before reaching a limit based on the carrying capacity of the land. Deviations above the modelled 95% confidence envelope represent periods of population growth in which carrying capacity is exceeded. This model has been used in a number of papers (cited in our paper) to examine population fluctuations using SPDs. We have added text at lines 214-215 and 219-222 to clarify this. We already note the limitations of the logistic model in lines 398-401.

The claim by the reviewer that 'post 4.2 ka BP event settlement and population were both successful and multiple times greater across this region' is not relevant in assessing the results of the logistic model, since these could also be phases of overshoot. More importantly, this claim is not supported by the data. Some individual surveys, notably around Tell Leilan, do experience substantial increases in settlement during the Middle Bronze Age, but at a regional scale rural settlement densities are comparable to those of the pre-4.2k phase and urban sites decline in density (see Figure 7 in our paper). Elsewhere (Lawrence et al. 2016, PLOS, Lawrence et al. 2017, Quat Int, Palmisano et al., in press, Quat Sci Rev) we have shown that population remains relatively low until the middle of the Iron Age in the Eastern part of our study area, and even later in the Western part. By this point very different technological and socio-political formations are in play.

Deviations above the modelled 95% confidence envelope represent periods of population growth in which carrying capacity is exceeded. This model has been used in a number of papers (cited in our paper) to examine population fluctuations using SPDs. We have added text at lines 214-215 and 219-222 to clarify this. We already note the limitations of the logistic model in lines 398-401.

The authors claim that post 4.2 event settlement that exceeds their carrying capacity estimate for regional population and agro-production, and which invalidates their entire argument, is not relevant --because it could be “overshoot”, i.e., exceeds their carrying capacity estimate (!).

Their erroneous citation of Ur 2010 for Akkadian population densities not exceeded until the 20th century AD ignores the Ur (2010: 159) comparison of the North Jezira Wilkinson-Tucker MB/OB period (ca. 1950-1700) population density at 0.088 sites/km and “only slightly lower” than the 0.095 sites/km density of the Leilan Region Survey –both much greater than the Akkadian period density. In other words, and as stated in the earlier review of this manuscript, the entire logic and thesis of this manuscript is disproven by the two published north Mesopotamian surveys which document larger populations in the post- Akkadian MB/OB periods than were occupying the same agricultural territory with the same agricultural technology at the 2200 BC regional collapse.

The authors also erroneously claim that:

Ll 410-411 “However, areas such as Western Syria show continuity 410 of settlement, and even substantial 411 urban growth, over the 4.2kya time window.” NO FOOTNOTE

There is no “continuity of settlement in areas such as western Syria and even substantial urban growth”: Umm el Marra (Schwartz 2014), Rawda (Brochier 2020), Titrish (Algaze and Pournelle 2005), Ta’yinat (Manning 2020), Ni’aj (Falconer 2020) are each abandoned at 2200 BC. Even Ebla, now Archaic Palace-filled with water cisterns, shrinks (Weiss 2000; Matthiae 2020).

7 . Takes SPDs as representative sample of human activity across time and space, and mysteriously of population, when SPDs only reflect archaeologists’ skewed 14C sampling, if any, across time and space (Palmisano et al 2017 lists all the errors in using SPDs), that is, "a hopeless endeavor" (Torfing 2015).

Palmisano is one of the authors of our paper. His 2017 paper cited by the reviewer does outline several criticisms of the SPD approach, but argues that SPDs of C14 dates are still a useful proxy when appropriately contextualised and compared with other proxies for population. This is precisely what we do in our paper. We acknowledge the limitations of the SPD approach in lines 103-112, including all criticisms outlines by Palmisano 2017. Here we also cite several recent papers both within and adjacent to our study region which find a good correlation between SPDs

We feel that a blanket assertion that ‘SPDs only reflect archaeologists’ skewed 14C sampling’ is not sustainable. We can support this claim through statistical examination of the relationship between SPD and landscape survey derived population curves, both within our paper and others cited in it covering parts of our study region and adjacent areas. More generally, SPDs have been widely used for two decades in several regions of the world, including in several papers published by PLOS.

We note that the quotation from Torfing 2015 is from a paper on the use of SPDs in European prehistory. The full quote reads: ‘For the Mesolithic–Neolithic transition and the Neolithic in general, this seems, to the present author, a hopeless endeavour’. Since we are dealing with a different period and region we do not feel this criticism is relevant here.

…growth we use as a null model. The model assumes that the long term growth rate of pre-industrial populations fits a logistic curve. The logistic population curve assumes population gradually increased before reaching a limit based on the carrying capacity of the land.

The logic of deploying a labile carrying capacity (based upon any number of models of regional agro-production functions and population estimates) is revealed in the authors' statement above:

the carrying capacity is a "function of the logistic model" and the logistic population curve is "based on the carrying capacity of the land." (!)

"Good correlation with SPDs"? The authors manuscript states:

"The population spikes after 4,000 cal BP present in the survey dataset are also less 329 marked in the urban dataset (Fig 6). *As with the survey dataset, all correlations between the SPD 330 and urban dataset improve if the analysis is performed only from 6,000-4,000 cal BP (Table 6), 331 suggesting the SPD is not capturing fluctuations in settlement during the period after 4,000 cal BP 332 as well."*

"After 4,500 cal BP, total rural settlement starts to decline while urban site numbers and the SPD 346 continue upwards. Both the urban and SPD datasets peak between 4,400 and 4,200 cal BP, after 347 which there is a rapid decline in the SPD over only around 100 years, followed by a slower paced 348 decrease. Urban site counts also fall from 4,200 cal BP but this is much more gradual, and the 349 overall numbers are fairly stable until the end of the Middle Bronze Age at 3,600 cal BP. *This 350 difference between the SPD and urban site datasets could be the result of differences in 351 chronological precision, such that a short period of reduced activity is visible in the relatively 352 chronologically sensitive SPD but is not evident in chronological attributions reliant on ceramic 353 changes*. However, if this were the case we might expect the SPD to recover into the Middle 354 Bronze Age (4,000-3,600 cal BP) when urban site counts remain high and rural sites experience a 355 second peak. *The fact that the SPD does not capture this may be due to research biases towards 356 excavation at Early Bronze Age sites and phases within sites, and the fact that several large Middle 357 Bronze Age site were excavated before radiocarbon dating was routinely employed."*

Illustrates how high resolution archaeological and paleoclimate data can be reconstructed into low resolution data, and thereby deployed to ambiguously support an otherwise disproven thesis.

As explained above (response to points 3 and 4), we do not use paleoclimate data in this paper and have added text clarifying this in lines 82-84. We also do not reconstruct high resolution data into low resolution data. In fact, the modelling techniques we have applied to the settlement data provide modelled values at an increased resolution. As explained above (response to point 1), the binning technique applied to the radiocarbon dates does not impact the resolution of the dataset in the manner suggested by the reviewer and we have added text clarifying this at line 194.

In summary, the fundamental facts of late third-early second millennium BC Mesopotamian archaeology and history are misrepresented, ignored, or distorted within a thin skein of statistics to support an otherwise disproven thesis.
