# Supplementary material for: Collapse and continuity: A multi-proxy reconstruction of settlement organization and population trajectories in the Northern Fertile Crescent during the 4.2kya Rapid Climate Change event
Source: PLoS One. 2021 Jan 11;16(1):e0244871. doi: 10.1371/journal.pone.0244871 (PMC7799814; doi:10.1371/journal.pone.0244871)
Supplement: Attachment — Submitted filename: Lawrence et al_Response_to_Reviewers.docx [file pone.0244871.s002.docx]

2^nd^ November 2020

Dear Professor Biehl,

We are very grateful to you and to the two reviewers who examined our manuscript entitled “Collapse and Continuity: A multi-proxy reconstruction of settlement organization and population trajectories in the Northern Fertile Crescent during the 4.2kya Rapid Climate Change event”. Below we respond (in plain text) to the points raised by each reviewer (reproduced in italics) and outline changes made to the text of the article as a result (in bold text). Where line numbers are mentioned these refer to the revised manuscript including track changes.

Reviewer 1

*1. Blurs high resolution 14C chronology in multiple regions into arbitrary 100 year bins, when the events and processes described are measurably visible at AMS-calibrated Oxcal-regulated and Bayes- manipulated decadal levels;*

The technique used and described in the paper does not blur chronological dates and does not assign individual dates to 100 year bins. We use an established technique (see citations in the paper) to mitigate the impact of single site phases with multiple radiocarbon dates close together on our curves. This uses 50 year bins (not 100 year bins) and is only applied to sites where there are multiple C14 dates within 50 years of one another (as explained in lines 190-192). Where we have multiple dates within 50 years the uncalibrated radiocarbon age and error of all dates are averaged, and we then calibrate the averaged date rather than multiple dates. We want to stress that this technique is only applied to a small number of phases at a small number of sites where there are multiple dates within 50 years. All other dates contribute to the curves at the resolution of the published date, and the graphs in the relevant figures are at a yearly resolution. **We have added text clarifying this at line 194**, in addition to the explanation at 189-193 which covers the above points.

Although not explicitly stated, we understand the reviewer to be concerned that the binning technique we use will mask very short-term fluctuations in the curve which can be attributed to the effects of the 4.2k event. We hope that the fact that we are applying a 50 year rather than 100 year bin will mitigate this concern, along with the clarification that we are only applying this to a limited number of sites. More fundamentally, the effect on the dataset is not to blur or coarsen it, but to smooth fluctuations resulting from sampling biases (something which the reviewer raises as important in point 7 below). We would also note that at a regional scale the duration of the 4.2k event is considered to be centennial rather than decadal (as we state in lines 79-81, with citations), meaning the resolution of our C14 dataset should be sufficiently precise to identify fluctuations resulting from it. In fact, as we state in lines 348-350 and show in Figures 2 and 7, we can see a rapid decline at 4.2k over only 100 years. We argue in lines 351-355 that it is the increased precision of C14 dates relative to other proxies which allows us to ‘see’ the event in this dataset.

*2. Misses Spain, France, Italy, Greece, Palestine, Egypt, Indus and East Asia societal collapses synchronous at 4.2 ka BP with northern Levant and northern Mesopotamia ("northern Fertile Crescent");*

Our paper does not pretend to include all of the regions where the 4.2kya event has been considered to have an impact, and we state the location of the study region in the title, abstract and introduction. We also acknowledge that the event is considered to have impacted societies beyond out study region (lines 55-56, 85-88) and provide several citations to support this. As we state in lines 85-92, the goal of our study is to investigate the event at a regional scale using empirical data as opposed to synthetic comparison, and the area we have chosen is based on a shared physical geography. A future project might compare all of the regions described by the reviewer using archaeological data but this would require years if not decades of data collection. While this work is undertaken, we feel regional analyses can still advance the debate on this topic, as Reviewer 2 suggests.

*3. Misses westerlies, ISM, and EASM synchronous, high resolution, abrupt 4.2 ka BP event;*

As stated in lines 66-68, the goal of this paper is to examine population and settlement organisation during the period of the 4.2kya event. We take it as fact that the event occurred in some form, and briefly explain the state of the debate on its magnitude and duration with appropriate citations (lines 71-82). As such we do not go into detail on the climate forcing mechanisms, such as the westerlies, Indian Summer Monsoon (ISM) and East Asian Summer Monsoon (EASM) shifts mentioned by the reviewer, which may account for the climate event itself. We are aware of these mechanisms, and cite relevant literature which deals with them, including our own work (Cookson et al. 2019), but we do not feel that further discussion is relevant to the goals of the paper. **We have added text clarifying that we are not dealing with the climate proxies here in lines 82-84 and an additional date range in line 80**.

*4. Misses high resolution northern Levant and north Mesopotamian region proxy data for abrupt, century-scale, 4.2 ka BP event;*

We are unsure what the reviewer is referring to here, but assume they mean climate proxy data. If this is the case then as above we stress that our paper is not addressing the climate proxy data, although we do cite several overview papers on climate proxy data derived from natural archives (speleotherms, pollen, formanifera etc) in the region. **We have added text clarifying this in lines 82-84 and an additional date range in line 80**. If the reviewer is referring to specific proxy data for another variable then we would be happy to address this concern if provided with more detail.

*5. Takes inexpert, pop archaeology, opinions (e.g., Middleton), lacking any data, for facts of Akkadian collapse;*

We cite multiple sources on the collapse of the Akkadian Empire, all of which come from peer reviewed journal articles, books and edited volumes. None of these would be considered popular archaeology. The two citations of Middleton are from a Cambridge University Press single author volume and a chapter in an edited volume. The CUP volume is not a popular science volume and was (favourably) reviewed in several academic publications including the European Journal of Archaeology (Smart 2018) and the American Journal of Archaeology (Tainter 2018) as an academic work. The chapter cited comes from a volume edited by Eustathios Chiotis which is also not a popular science book. All contributions to this volume were peer reviewed and the authors are all established academics. As such we feel this comment is unwarranted. We also note that the reviewer does not identify any substantive criticisms of our treatment of the Akkadian Empire in the paper. Without knowing what facts are incorrect we cannot amend the manuscript.

*6. Guesses at testing of regional societies' carrying capacity limit at end 3rd mill., when post 4.2 ka BP event settlement and population were both successful and multiple times greater across this region;*

The carrying capacity limit is not a guess, it is a function of the logistic model for population growth we use as a null model. The model assumes that the long term growth rate of pre-industrial populations fits a logistic curve. The logistic population curve assumes population gradually increased before reaching a limit based on the carrying capacity of the land. Deviations above the modelled 95% confidence envelope represent periods of population growth in which carrying capacity is exceeded. This model has been used in a number of papers (cited in our paper) to examine population fluctuations using SPDs. **We have added text at lines 214-215 and 219-222 to clarify this**. We already note the limitations of the logistic model in lines 398-401.

The claim by the reviewer that ‘*post 4.2 ka BP event settlement and population were both successful and multiple times greater across this region’* is not relevant in assessing the results of the logistic model, since these could also be phases of overshoot. More importantly, this claim is not supported by the data. Some individual surveys, notably around Tell Leilan, do experience substantial increases in settlement during the Middle Bronze Age, but at a regional scale rural settlement densities are comparable to those of the pre-4.2k phase and urban sites decline in density (see Figure 7 in our paper). Elsewhere (Lawrence et al. 2016, PLOS, Lawrence et al. 2017, Quat Int, Palmisano et al., in press, Quat Sci Rev) we have shown that population remains relatively low until the middle of the Iron Age in the Eastern part of our study area, and even later in the Western part. By this point very different technological and socio-political formations are in play.

*7. Takes SPDs as representative sample of human activity across time and space, and mysteriously of population, when SPDs only reflect archaeologists’ skewed 14C sampling, if any, across time and space (Palmisano et al 2017 lists all the errors in using SPDs), that is, "a hopeless endeavor" (Torfing 2015).*

Palmisano is one of the authors of our paper. His 2017 paper cited by the reviewer does outline several criticisms of the SPD approach, but argues that SPDs of C14 dates are still a useful proxy when appropriately contextualised and compared with other proxies for population. This is precisely what we do in our paper. We acknowledge the limitations of the SPD approach in lines 103-112, including all criticisms outlines by Palmisano 2017. Here we also cite several recent papers both within and adjacent to our study region which find a good correlation between SPDs and other population proxies derived from settlement data. In the results section we use Pearson’s correlation coefficient to compare between the SPD and settlement derived proxies. We describe under what conditions we find statistically robust correlations between the different proxies and show that for the period between 6,000 and 4,000 the correlations between settlement derived proxies and SPDs are robust. In the discussion section we contextualise the relationship between SPDs and settlement datasets (e.g. lines 370-371, 376-377).

Overall we feel that SPDs, although, like all proxies (including landscape survey data), imperfect, can provide insights into past population if handled appropriately. We also feel that a blanket assertion that ‘*SPDs only reflect archaeologists’ skewed 14C sampling*’ is not sustainable. We can support this claim through statistical examination of the relationship between SPD and landscape survey derived population curves, both within our paper and others cited in it covering parts of our study region and adjacent areas. More generally, SPDs have been widely used for two decades in several regions of the world, including in several papers published by PLOS.

We note that the quotation from Torfing 2015 is from a paper on the use of SPDs in European prehistory. The full quote reads: ‘For the Mesolithic–Neolithic transition and the Neolithic in general, this seems, to the present author, a hopeless endeavour’. Since we are dealing with a different period and region we do not feel this criticism is relevant here.

*Illustrates how high resolution archaeological and paleoclimate data can be reconstructed into low resolution data, and thereby deployed to ambiguously support an otherwise disproven thesis.*

As explained above (response to points 3 and 4), we do not use paleoclimate data in this paper and **have added text clarifying this in lines 82-84**. We also do not reconstruct high resolution data into low resolution data. In fact, the modelling techniques we have applied to the settlement data provide modelled values at an increased resolution. As explained above (response to point 1), the binning technique applied to the radiocarbon dates does not impact the resolution of the dataset in the manner suggested by the reviewer and **we have added text clarifying this at line 194.**

Reviewer 2

*The approach presented in this manuscript is a welcome addition in archaeology, especially the kind of research that combines long-term climatic changes and regional settlement patterns. Although the research area encompasses several different climate zones and the approach may distort valuable sub-regional variations in climatic and cultural spheres, we need to test supra-regional approaches and analyses of archaeological data.*

We acknowledge that further investigation of sub-regional trends would be extremely interesting (and in fact have published longer term trends by region in PLOS, Lawrence et al. 2016) and we acknowledge this in the paper (e.g. lines 406-413, 432-433). However, given the volume of data involved we feel that detailed discussion of regional trends would be too much for this paper.

*I have to stress however, that 4.2 is far from being agreed upon as a global event as authors state in line 76!*

We agree with this statement but wanted to convey the fact that the International Commission on Stratigraphy have recently established the 4.2k event as a boundary marker for the end of the Middle and beginning of the Late Holocene, suggesting consensus in at least one field. **We have amended the sentence to clarify this point (lines 76-78)**.

Additional Review Comments

We have addressed the copyright concerns raised regarding Figures 1,2 and 6. All figures were made using freely available and reproducible materials. **The source material is now described in the caption of each figure, and all sources allow for academic reproduction under a CC BY 4.0 licence** (see USGS website and <https://gadm.org/license.html>). **We have also deleted some additional line breaks in the bibliography.**

We understand that the changes we have made to the manuscript may not be considered substantial. However, we feel that several of the criticisms outlined by Reviewer 1 are either due to misunderstandings which can be resolved through clarification in our manuscript (e.g. inclusion of climate data), or are so fundamental to the goals of the paper that they cannot be accommodated (e.g. objection to the use of SPDs as a proxy for population). We would be very grateful for a steer from the editor if there are specific changes which need to be made, and of course we would be happy to make them. If further reviewers are required we would also be happy to engage with their comments.

Once again, we thank the reviewers and the editor for their time and effort.

Yours sincerely,

Dr Dan Lawrence,

Associate Professor in the Archaeology of the Ancient Near East

Durham University
