# Supplementary material for: Collapse and continuity: A multi-proxy reconstruction of settlement organization and population trajectories in the Northern Fertile Crescent during the 4.2kya Rapid Climate Change event
Source: PLoS One. 2021 Jan 11;16(1):e0244871. doi: 10.1371/journal.pone.0244871 (PMC7799814; doi:10.1371/journal.pone.0244871)
Supplement: Attachment — Submitted filename: Lawrence et al_Response_to_Reviewer.docx [file pone.0244871.s004.docx]

13^th^ December 2020

Dear Professor Biehl,

We are very grateful to you and to the reviewers who re-examined our manuscript entitled “Collapse and Continuity: A multi-proxy reconstruction of settlement organization and population trajectories in the Northern Fertile Crescent during the 4.2kya Rapid Climate Change event”. Below we respond (in plain text) to the points raised by Professor Zerboni (Reviewer 3, reproduced in italics) and outline changes made to the text of the article as a result (in bold text). Where line numbers are mentioned these refer to the revised manuscript including track changes. We have not responded to the comments from Reviewer 1 since we were instructed to address only Reviewer 3 comments. However, we have looked at Reviewer 1’s comments and would be happy to address any of their points if required.

Reviewer 3

*Maybe some more sentences at the beginning of the discussion explaining the concept of carrying capacity of the environment would help the reader to understand the conclusion of the discussion.*

**We have added sentences explaining the concept of carrying capacity and its relationship with the logistic growth model at lines 401-406. We have also added a reference to a similar use of this method.**

*The story this paper tells of continuity and adaptation; in fact, I would like to understand whether this is a case of population relocation and/or major change in land use. As you are considering the hinterlands of the main Akkadian capital (which is the crucial area where to investigate social instability and collapse), can you detect in this phase a shift from agricultural to pastoral vocation of villages? Do you have evidence to establish whether a shift in land use as a tuning factor or a consequence of the social reorganisation?*

This is a very interesting point, and one that has not been addressed in detail by our paper or others. There is also a complicating factor here in that the phase immediately after the 4.2k event is when true nomadic pastoralism likely emerged in our study region. Because this form of existence leaves very limited material traces in the archaeological record, it is difficult to distinguish between population relocation and a land-use shift to mobile pastoralism – both result in an absence of evidence for sites in the landscape. In our current ERC project we are hoping to address this by gathering the limited archaeobotanical and zooarchaeological data available from excavations in the region to see if changes in animal exploitation are visible in the periods before, during and after the 4.2kya event. We will also use this so reconstruct land use through Species Distribution Modelling. However, we do not have these data as yet. **For this paper we make reference to land use change in a new section in the discussion (lines 389-400) and discuss the limited evidence currently available for this (including new references).**

*In the discussion you discuss the decrease in productivity related to increased exploitation of natural resources. Also this is a major point that can be explained more in details. There is a number of modern and paleo case studies illustrating how overgrazing under unstable climatic conditions lead to the disruption of ecological equilibrium and thus to the disruption of non-flexible land use strategies. You can find some example cited in Zerboni and Nicoll 2019 Geomorphology.*

We are very grateful to Professor Zerboni for pointing us to this literature. **We have added a section in the discussion (lines 389-400) which deals with overgrazing and the (limited) evidence available for this in our region.** This includes evidence from our preliminary work on land use reconstruction and several other new references. As mentioned above, overexploitation, extensification and intensification are topics we intend to address in future papers once the data collection phase of our current project provides sufficient evidence to make firm statements on this.

*Finally, In the conclusion, I would underline the concept of continuity, which is the major take-home message of the manuscript.*

**We have added a sentence emphasising this to the conclusion (lines 445-447) and an additional reference on continuity of occupation in Western Syria in particular.**

Additional Data

Between submission and the return of the manuscript we have enhanced our dataset by including a very small number (10) of additional radiocarbon dates, taking the total used to 963. For this revised version we have rerun all analysis to take these into account. This has no material impact on the results or conclusions of the paper. All results remain significant or not significant as previously stated. **Changes are highlighted in Tables 1-4, and there are very minor shifts in the SPD curves in the Figures 2-5 and 7**. The figure changes are close to invisible to the naked eye but we include new figures for the sake of completeness. **We have also now added the C14 dataset to the Supplementary Materials.**

Once again, we thank the reviewers, especially Professor Zerboni and the editor, for their time and effort.

Yours sincerely,

Dr Dan Lawrence,

Associate Professor in the Archaeology of the Ancient Near East

Durham University
